# Integrating care across the life course supports goal-aligned end-of-life care

**DOI:** 10.1038/s43856-026-01807-z

**Published:** 2026-07-20

**Authors:** M. Courtney Hughes

**Affiliations:** School of Preventive and Applied Health, Northern Illinois University, DeKalb, IL USA

**Keywords:** Palliative care, Public health

## Abstract

As global populations age and lifespan increases, health systems too often conceptualize “healthy aging” and “end-of-life care” as distinct phases. Yet emerging evidence from geroscience, palliative care, and health systems science suggests that aging and dying lie on a continuum of adaptation and care. This Perspective proposes a framework that treats system-supported, goal-aligned end-of-life care as a public health outcome of integrated life-course care and argues for system redesign to introduce earlier palliative approaches, align care models across the life-course, and reduce health disparities. This article outlines key mechanisms, evidence for integration, a conceptual model, and implications.

## Abstract (web summary)

Hughes argues that goal-aligned end-of-life care should be understood as a public health outcome of integrated life-course care. It proposes a framework that unifies aging and dying as a continuum, highlighting opportunities for earlier palliative integration, community-based support, and technology-enabled continuity.

## Introduction

The 21st century is defined by aging. Globally, the number of people aged 65 and older is projected to double from 761 million individuals in 2023 to 1.6 billion individuals in 2050 and will account for 1 in 6 people globally^1^. The World Health Organization (WHO) defines *healthy aging* as “the process of developing and maintaining the functional ability that enables well-being in older age,” emphasizing the interaction between intrinsic capacity and enabling^2^. Building on this foundation, the United Nations launched the Decade of Healthy Aging (2021–2030) to advance global collaboration around longer, healthier lives^3^.

Despite this progress, in health systems practice, aging care (focusing on prevention, functional maintenance, and chronic disease) and end-of-life care (focusing on palliation, dying, and bereavement) operate in largely separate silos. This separation often leads to missed opportunities, as the mechanisms that support resilience and adaptation in aging are also relevant to goal-concordant dying and end-of-life transitions, which can benefit from earlier system-based planning and care alignment. The recent Lancet Commission on the Value of Death draws attention to how death and dying are under-integrated in health-systems design^4^.

This Perspective argues that system-supported, goal-aligned end-of-life care should be treated as part of the full life-course, which means rethinking system architecture, clinical models, and policy frameworks. The article proceeds in five sections: (1) a review of how current care models separate aging and dying; (2) emerging integrated models and evidence; (3) a proposed conceptual model with aging as a continuum across the life-course; (4) technology as a mechanism for continuity, and (5) policy and societal implications. Importantly, this framework is not intended to prescribe a normative model of aging or dying, but to support conditions for value-concordant, relational, and equitable care. Additionally, in this article, “healthy aging” is understood as the maintenance of functional ability and adaptation within one’s context, while end-of-life care is evaluated based on the degree to which care is aligned with individual goals, values, and relationships, rather than predefined clinical or functional benchmarks.

## Fragmentation of Care: Aging vs Dying

Modern health systems evolved around acute care and disease specialization, not life-course continuity. In most countries, services for older adults are divided into chronic disease management, geriatric care, rehabilitation, and long-term care, while dying is handled through hospice and palliative units. The organizational boundaries such as health professional training and insurance billing codes create invisible walls between these stages^5,6^. The WHO’s healthy aging agenda emphasizes functional ability, intrinsic capacity, and environments (including supportive communities) as central to aging well^2^. However, there is little explicit integration of system-supported, goal-aligned end-of-life care or transition planning within that agenda.

The distinction between aging and dying is more a product of system design than of human biology. From a biological perspective, both processes involve adaptation, loss of reserve, accumulation of multimorbidity, and eventual transition to end-of-life. Geroscience shows that mechanisms of aging (e.g., inflammation, cellular senescence) also correlate with vulnerability to dying^7,8^. Systemically ignoring that overlap creates inefficiencies. Palliative care is specifically designed to address needs across the trajectory of serious illness, including symptom management, communication, and care planning, and thus already provides a framework for bridging the divide between aging and end-of-life care^9^. However, in practice, access remains uneven and often delayed, limiting its ability to function as a continuous support across the life course. For example, individuals with advanced heart failure frequently require intensive symptom management, home-based diuretic adjustment, and close monitoring to prevent exacerbations, yet access to such home-based palliative services remains limited in many settings. Expanding these models could reduce hospitalizations and improve continuity of care.

The result is a discontinuity in care. Patients aging with multimorbidity often experience abrupt transitions from “curative” to “palliative” phases without adequate preparation or coordination. Furthermore, studies show that earlier care planning intervention would allow individuals to express their wishes and improve quality of life^10–12^. When palliative care begins late, patients experience more hospitalizations, higher costs, and poorer quality of death^13^. Furthermore, a substantial proportion of patients fall into an “in-between” category: they are neither acutely ill nor imminently dying, yet live with complex, progressive conditions requiring ongoing symptom management and care coordination. Current health systems are poorly structured to meet these needs, often resulting in reactive, crisis-driven care rather than anticipatory, continuous support.

Separation of aging and dying also contributes to disparities. Older adults who age with poor functional support may face abrupt transitions to dying with less planning, more symptom burden, and fewer supports^14,15^. Those with limited access to palliative care services or fragmented care are less likely to experience system-supported, goal-aligned end-of-life care^16^. Low-income, rural, and racially marginalized populations are less likely to receive hospice services and more likely to die in hospitals rather than at home^17^. The Lancet Commission on the Value of Death emphasizes how structural determinants, such as income, gender, and geography, shape how people die^4^.

## Integrated Models: Evidence and Opportunity

There is growing evidence that introducing palliative care earlier in the trajectory of chronic disease or frailty improves quality of life and reduces symptom burden^18,19^. For example, in older adults with heart failure or chronic obstructive pulmonary disease, early palliative integration reduces hospitalization and improves care planning^12,20,21^. Similar results have been observed in dementia care, where early identification of palliative needs supports caregiver well-being and reduces crisis-driven transitions to acute care^22^. These approaches blur the traditional division between aging care and end-of-life care. This life-course framing aligns with the Care Planning Umbrella model, which conceptualizes advance care planning and serious illness communication as processes that span healthy living, chronic illness, frailty, and end-of-life, rather than discrete, stage-bound interventions^12^.

Some health systems have developed models of aging through to end-of-life care in a manner that align community care, primary care, geriatrics, and palliative care into a continuum. For instance, age-friendly communities emphasized in the Decade of Healthy Aging include long-term care, community support, and end-of-life-friendly design^3^. Japan, for example, has pioneered a comprehensive model known as the Community-Based Integrated Care System (CBICS), instituted in response to an unprecedented rate of population aging. The government has aimed to establish a structure that enables older adults to spend their remaining years living in the community rather than institutional settings, while guaranteeing access to health care, long-term care, preventive services, housing, and social supports^23^. Under CBICS, accessible services are designed at the neighborhood level and enable older persons with chronic disease or increasing care needs to transition seamlessly from preventive to rehabilitative to palliative supports without being confined to institutional pathways^24,25^. Japan’s approach encourages early identification of physical and cognitive decline and coordination between medical and community-based programs^26^.

In the U.S., Kaiser Permanente’s Life Care Planning incorporates palliative principles into chronic-care management, triggering early referrals based on predictive analytics for frailty. This model emphasizes brief, iterative discussions that address patients’ evolving needs and leverages the electronic health record to coordinate care. By normalizing serious illness conversations throughout patients’ lives, this model promotes consistency and readiness^27^.

Integrated care models have a potential equity dividend. When transitions from aging to dying are planned, support is community-based rather than reactive and resources are aligned across the timeline^28–30^. Community-based networks, such as faith organizations, neighborhood volunteers, and peer navigators, fill gaps left by formal systems. The Value of Death Commission calls for the ‘health-care death system’ to be replaced by a relational, community-based system that spans life’s end^4^. Early palliative care reframes dying not as failure but as a predictable phase requiring anticipatory support. Embedding palliative principles like care planning and symptom management within routine geriatric or chronic-care encounters helps normalize end-of-life discussions and align care goals. In this sense, the continuum proposed here does not introduce a new clinical paradigm but rather makes explicit what palliative care has long advocated: that support for serious illness, symptom management, and goal-concordant care should be integrated earlier and more consistently across the life course. The contribution of this framework lies in reframing these principles at a systems level, emphasizing their application beyond specialty settings and across population health and policy domains.

## Toward an Integrated Continuum of Aging and End-of-Life Care

**Fig. 1:**
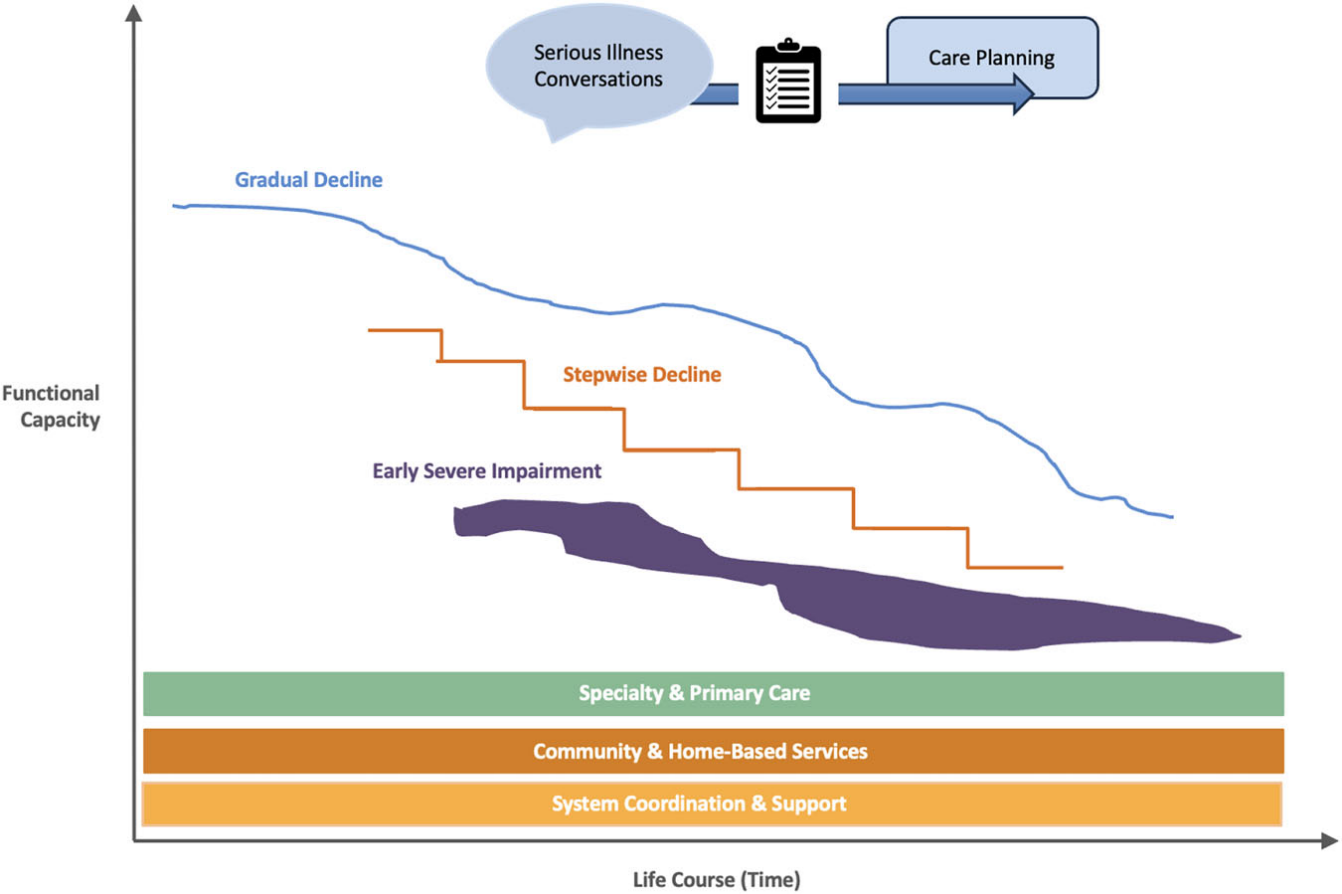
Heterogeneous Aging and End-of-Life Trajectories with Integrated System and Community Support Across the Life Course.

Rather than viewing aging and dying as distinct episodes, a life-course lens highlights them as overlapping phases characterized by changing goals, capacities, and support needs. This perspective reframes the trajectory from midlife to end-of-life as a continuum of adaptation, where biological decline and shifting caregiving demands unfold gradually, not abruptly. Figure 1 presents a conceptual model of heterogeneous aging and end-of-life trajectories, illustrating that changes in intrinsic capacity are not linear or uniform across individuals. Trajectories may include gradual decline, stepwise deterioration with periods of recovery (e.g., heart failure), or early functional limitation due to chronic illness. The model emphasizes that, despite this variability, clinical care, community support, and care coordination should be continuously integrated across the life course, rather than introduced only at the end of life. The arrow at the top of Fig. 1 represents this continuity. Serious illness conversation informs care planning across the life course, consistent with the Care Planning Umbrella model^12^. Rather than depicting a single pathway, this model highlights that individuals may enter periods of higher support need at different points in the life course, reinforcing the importance of flexible, anticipatory, and goal-aligned care models.

Crucially, the continuum emphasizes anticipatory alignment: the earlier systems identify changes in functional trajectories, the more effectively they can coordinate supports. This includes proactive care planning, early palliative assessment, community mobilization, and integration across clinical silos. When these processes occur upstream, individuals and families experience transitions that are smoother and better aligned with personal values.

This framing also elevates the role of communities. Families, neighborhoods, faith networks, and lay navigators operate as stabilizing forces throughout the continuum, particularly during the aging-to-dying transition. Their involvement reflects the Lancet Commission’s argument that dying well is relational, not solely biomedical^4^. This supports family caregiving and cultural practices often overlooked by traditional health-systems design that have been shaped by biomedical and provider-centric frameworks. These traditional designs have prioritized clinical services, organizational performance, and disease management. Donabedian’s structure–process–outcome model, for example, has been foundational in defining quality through measurable clinical inputs and outcomes^31^. Subsequent reform efforts, including the Chronic Care Model, explicitly recognized the importance of self-management and community resources in supporting individuals with long-term conditions^32^. However, in practice, the health system has remained the primary organizing agent, with community and family support positioned as complementary rather than central. As a result, commonly used models offer limited guidance for supporting transitions into dying, when functional capacity declines and reliance on informal caregiving and community networks intensifies^4^. Table 1 contrasts traditional, disease-centered health system structures with the life-course integrated continuum proposed here. Taken together, these elements illustrate a conceptual shift away from episodic, disease-centered care toward a relational, life-course architecture that attends to both living well and experiencing system-supported, goal-aligned end-of-life care.

**Table 1:** From Disease-Centered Models to Life-Course Integrated, Community-Engaged Care

Dimension	Traditional Health Systems	Palliative Care (Existing Model)	Life-Course Integrated Continuum (This Framework)
Orientation	Disease-specific, episodic	Serious illness–focused, interdisciplinary	Life-course, population health–oriented
Timing	Triggered by acute events	Introduced during serious illness (often late)	Initiated early and sustained across aging, chronic illness, and end of life
Scope of Care	Cure, disease management	Symptom management, communication, care planning	Integration of prevention, adaptation, care planning, and end-of-life support
Care Planning	Episodic, often late	Structured care planning and serious illness communication	Longitudinal, iterative care planning across life stages (Care Planning Umbrella^12^)
Workforce	Specialty silos	Interdisciplinary teams (palliative specialists)	Primary + specialty integration; primary palliative care across workforce
Community Role	Minimal	Supportive, often peripheral	Central: community, family, and social systems integrated with care
System Integration	Fragmented across settings	Coordinated within palliative programs	Integrated across clinical, community, and policy systems
Payment Model	Fee-for-service, procedure-based	Limited reimbursement; often episodic	Requires sustained investment in home and community-based services, caregiver supports, and coordination infrastructure
Equity Implications	Variable access	Uneven access to palliative services	Explicit focus on equitable access to community-based supports and caregiving resources
Outcome Focus	Mortality, utilization	Quality of life, symptom burden	Goal-aligned care, continuity, caregiver well-being, equity

It is important to note that reliance on community-based and family caregiving alone is neither sufficient nor equitable. The availability of community and family support varies widely across socioeconomic and cultural contexts, and without adequate financial and structural support, these systems may be strained or inaccessible. Sustainable implementation of community-centered models therefore requires explicit investment in home- and community-based services, caregiver supports, and coordination infrastructure.

## Policy and Societal Implications

Operationalizing the conceptual model in Fig. 1 requires clearer delineation of responsibility across system actors. Policymakers and payers play a central role in establishing the conditions for integration by aligning reimbursement, quality metrics, and regulatory frameworks with life-course continuity and goal-concordant care. Health systems and clinical organizations are responsible for redesigning care delivery to embed palliative principles earlier, integrate services across settings, and support interdisciplinary teams. Community-based organizations, including faith groups, volunteer networks, and lay navigators, serve as essential partners in providing relational continuity and culturally grounded support. The proposed model functions as a coordinating framework that aligns these actors around shared objectives of reducing fragmentation and improving patient and caregiver experience while policy and payment reforms create incentives for sustained collaboration. These partnerships are further motivated by shared system-level benefits, including reduced avoidable hospitalizations, improved patient and caregiver outcomes, and more efficient use of health system resources.

### Sequencing priorities and enabling macro systems

Sequencing matters because the ten dimensions in Table 1 cannot move at once. Payment model and workforce should be prioritized first because they set the conditions under which every other dimension can change. Reimbursement that compensates longitudinal, team-based work is needed for providers to sustain serious illness conversations across the life course, and primary palliative care competencies must be embedded across the broader workforce. Value-based payment pilots from the Centers for Medicare & Medicaid Services (CMS)^33,34^ and the implementation work of the Center to Advance Palliative Care illustrate how payment and workforce reforms can advance together^35,36^. With these foundations in place, care planning becomes operationally feasible, along with system integration and earlier timing of palliative engagement. The remaining dimensions, which concern community role, equity, and outcome measurement, depend on the maturation of these mid-level changes and require sustained investment in non-clinical infrastructure such as caregiver supports and lay navigation.

Responsibility for advancing each dimension is distributed across actors, and the partnerships between them are themselves part of the work. Payers and policymakers, including CMS and analogous national or regional payers, along with ministries of health and private insurers, hold the levers for reimbursement and quality metric alignment, and they can require or incentivize health-system partnerships with community organizations as a condition of payment. Health systems and clinical leaders, working with professional societies and academic training programs, can lead workforce redesign and embed palliative competencies into primary and specialty settings. Their operational link to community-based care runs through accountable care contracts and formal agreements with Area Agencies on Aging and other home- and community-based service providers, as well as embedded community health worker programs. Community-based organizations such as faith networks and lay navigator programs deliver relational continuity, and their lasting integration with health systems depends on shared governance and common data infrastructure, supported by contracting that funds ongoing collaboration rather than ad hoc projects. Public health departments can coordinate across these sectors and link clinical care to population-level supports. Patient and family advisory bodies should inform metric design and care model implementation, keeping individual values at the center of reform.

The continuum approach also depends on macro systems beyond health systems design. Long-term care financing, often separated from acute medical coverage, is a foundational precondition, particularly where home- and community-based services depend on out-of-pocket payments or means-tested eligibility^37^. Caregiver-supportive labor policies, such as paid family leave and workplace protections with adequate respite benefits^38^, matter alongside housing and transportation that enable aging in place^39^, as does broadband infrastructure capable of supporting telepalliative care^40^. Legal recognition of advance directives and portable medical orders^41^, together with clarity around surrogate decision-making across jurisdictions^42^, also forms part of the supporting macro architecture. Where these systems are absent or misaligned, the aging-to-dying continuum risks collision with structural barriers beyond the reach of health system reform.

### Redefining metrics of success

Healthy aging frameworks have traditionally emphasized functional ability, independence, and the maintenance of physical and cognitive capacity across the life course, while end-of-life metrics have focused more narrowly on mortality, health care utilization, and costs incurred near death. This bifurcation reinforces the conceptual and operational separation of aging and dying within health systems. Integrating these domains requires the development and adoption of indicators that extend beyond survival or service use to capture outcomes such as quality of dying, congruence between expressed preferences and care received, relational continuity, and family caregiver well-being. These measures better reflect lived experience during the aging-to-dying transition and align with emerging calls to view dying as a public health concern rather than solely a clinical event.

From a population health perspective, surveillance systems could incorporate “healthy dying” indicators analogous to Healthy Life Expectancy (HALE), capturing not only how long people live, but how well they are supported at the end of life. Early efforts in this direction demonstrate feasibility. For example, Finkelstein and colleagues’ cross-national comparison of quality of death employed a preference-based scoring algorithm to assess end-of-life care performance across countries, illustrating how multidimensional indicators can be operationalized at the system level^43^. Such approaches offer a foundation for comparative learning while highlighting gaps between policy intent and lived experience.

At the same time, the development of dying-related metrics must be approached with caution. There is substantial variation across countries, cultures, and religious traditions regarding what constitutes a “good death,” including differing values placed on autonomy, symptom control, family presence, spiritual care, and place of death^44^. Universal indicators therefore risk oversimplification if they fail to account for cultural plurality and contextual meaning. Furthermore, measurement frameworks should prioritize whether care reflects individuals’ expressed values, preferences, and priorities over time. Patient-reported outcome measures, such as assessments of whether individuals feel heard and understood in serious illness communication^45^, offer more context-sensitive approaches to evaluating care quality in palliative care settings. These measures capture relational and experiential dimensions of care that are central to patients and families but often overlooked in traditional health system metrics. A flexible measurement framework that combines core indicators with locally defined dimensions may be necessary to ensure that metrics of healthy aging and system-supported, goal-aligned end-of-life care remain both analytically robust and ethically grounded.

The ethical grounding of this approach rests on three complementary principles. First, respect for persons emphasizes the importance of aligning care with individuals’ values, preferences, and goals across the life course, including at the end of life^46,47^. Second, a relational ethics perspective recognizes that aging and dying are embedded within networks of family, community, and care relationships, and that well-being cannot be understood solely at the level of the individual^4^. Third, principles of justice require attention to inequities in access to resources, services, and supports that shape both aging and dying experiences^17,48^. Together, these perspectives support a flexible, pluralistic framework that does not prescribe a singular model of a “good death,” but instead seeks to create conditions under which diverse individuals and communities can experience care that is meaningful, culturally congruent, and equitably supported.

### System and workforce integration

Health systems must redesign training and reimbursement to encourage continuity. Cross-training geriatrics and palliative-care providers remains essential; however, given workforce constraints, there is a growing need to embed primary palliative care and geriatrics competencies across the broader clinical workforce, including primary care, specialty care, and community-based providers. Expanding these competencies enables earlier identification of needs, more consistent serious illness communication, and improved continuity across care settings. Additionally, embedding care planning into routine visits and leveraging nurse-led community models can reduce fragmentation. Many of these approaches, including embedding care planning in routine care and integrating serious illness communication, are well established within palliative care and hospice practice. The challenge lies not in their conceptual development, but in their consistent implementation across settings and earlier stages of illness.

Health systems must redesign both training and reimbursement structures to better support continuity across aging and dying. Current workforce models often reinforce specialty silos, with geriatrics, primary care, and palliative care operating in parallel rather than in coordinated pathways. Cross-training clinicians in geriatrics and palliative care competencies (e.g., symptom management, prognostication, and serious illness communication) can help bridge this divide and promote earlier, more integrated support for patients with complex needs. Embedding care planning and goals-of-care discussions into routine clinical encounters, rather than reserving them for moments of crisis, further normalizes anticipatory planning and aligns care with evolving preferences over time.

Equally important are payment and organizational reforms that recognize relational and longitudinal work. Fee-for-service reimbursement models often undervalue care coordination, family caregiver engagement, and interdisciplinary collaboration, discouraging providers from investing time in activities that support continuity. Alternative payment models that incentivize team-based care, longitudinal management, and community engagement may better align system priorities with patient and family needs. Nurse-led and community-based models, such as those involving community health workers and palliative nurse navigators^28,29,49^, offer promising approaches for reducing fragmentation, particularly for older adults with declining functional capacity. These models leverage trusted relationships and local knowledge to connect clinical care with social and community supports, reinforcing continuity across settings and transitions.

Equally critical is the recognition that clinical workforce redesign alone is insufficient without parallel investment in caregiving and community-based supports^50^. Family caregivers provide the majority of long-term and end-of-life care, yet existing policies to support them remain fragmented, underfunded, and inconsistently evaluated. A recent systematic review of U.S. caregiver policies identified key provisions that appeared in policies such as respite services, caregiver training, workplace protections, and care coordination, but also highlighted persistent gaps in access, equity, and implementation, as well as limited evidence on policy effectiveness^38^. Without adequate financing and integration of these supports into health system design, caregivers face substantial burdens, which in turn can compromise care quality and continuity.

Payment and policy structures must therefore extend beyond clinical services to include sustained investment in home- and community-based services, family caregiver supports, and coordination infrastructure. When these supports are absent or inadequately reimbursed, transitions become more crisis-driven and outcomes for patients and families worsen. Conversely, aligning reimbursement with community-based and caregiver-centered models can strengthen continuity and improve both patient and caregiver experience across the aging-to-dying continuum^28,29,51^. While some regions, including parts of South Asia, have longstanding traditions of family-based elder care, these models are shaped by specific cultural, social, and economic contexts and cannot be assumed universally. Health systems must therefore design supports that are adaptable to varying community structures and resource environments.

### Technology as an enabler

No contemporary discussion of healthy aging is complete without addressing the role of technology. As digital tools increasingly shape how individuals live, technology has become a potentially important layer of the aging-to-dying continuum rather than an optional add-on. Its influence spans from monitoring intrinsic capacity to supporting communication and care coordination. Remote monitoring tools, sensor systems, and digital health platforms allow clinicians and family caregivers to identify declining function earlier, prompting proactive planning rather than crisis-driven responses. Studies show that wearables and passive home sensors can accurately detect changes in mobility, sleep patterns, and physiological markers that predict frailty progression and hospitalization risk^52,53^. Such systems extend the reach of health care into the home and community, enabling “aging-in-place” models that align with older adults’ preferences and reduce reliance on emergency or institutional care. As the UN’s Decade of Healthy Aging emphasizes, technology can enhance intrinsic capacity when implemented with equity and usability in mind, improving continuity across health and social systems^3^.

At the same time, the role of technology in care is not unproblematic. Digital tools can fragment attention, introduce new forms of surveillance, and, in some cases, depersonalize interactions that are central to high-quality aging and end-of-life care. There is a risk that technology-mediated encounters may displace rather than deepen human relationships, particularly when efficiency is prioritized over presence^54,55^. These concerns are especially salient in palliative contexts, where relational continuity, trust, and meaning-making are foundational.

Digital tools also have the potential to create new pathways for meaningful, relational support during the transition to dying. Telepalliative care (the remote delivery of palliative care), for example, has been shown to improve symptom management, reduce burdensome end-of-life utilization, and improve patient and family caregiver satisfaction, particularly in rural or underserved regions where specialty services are scarce^56–59^. Technology can also enable enhanced information sharing. For example, electronic medical records can capture serious illness conversations over time, making them easily retrievable for clinicians across disciplines and settings^27^. Communication platforms facilitate earlier care planning discussions and offer scalable opportunities for peer and community-based accompaniment (key themes highlighted by the Lancet Commission on the Value of Death)^4,12,60,61^. Emerging technologies such as conversational agents and digital legacy tools may further support aging individuals and their families. When thoughtfully designed, technology becomes not a replacement for relational care, but a scaffold that intentionally strengthens, rather than substitutes for, human relationships. The benefits of technology are therefore contingent on its design and implementation: tools that are user-centered, minimally intrusive, and explicitly oriented toward enhancing communication and coordination are more likely to support relational care, whereas poorly designed or workflow-driven technologies may exacerbate fragmentation and depersonalization.

It is crucial to note that technological optimism must be tempered by equity: older adults with low digital literacy or limited broadband access risk exclusion. Digital health innovations often assume access to reliable connectivity, appropriate devices, and the skills required to engage with platforms, assumptions that do not hold uniformly across socioeconomic, rural–urban, racial, or disability contexts. Without intentional design and implementation strategies, technology-enabled models of care may inadvertently widen existing disparities by privileging those with greater digital and social resources. They may also create relational inequities, where those with limited digital access or literacy experience not only reduced access to services, but diminished opportunities for meaningful engagement with care teams. Equity-oriented approaches therefore require investments in digital infrastructure, user-centered design, and support for family caregivers and community intermediaries who can help mediate access and interpretation. This can help ensure that technological advances enhance rather than undermine continuity of care across the aging-to-dying continuum.

### Community empowerment

Embedding care within social networks aligns health systems more closely with the cultural and relational dimensions of aging and dying. Families, faith communities, neighborhood organizations, and lay volunteers often provide continuity and practical support that formal services cannot fully replicate. “Compassionate communities” refer to a public health approach that recognizes dying as a shared social process and seeks to mobilize community assets rather than relying solely on professionalized medical care to support people and families at the end of life^62^. Initiatives grounded in this approach demonstrate that when dying is reframed as a collective responsibility rather than an exclusively medical event, fear and isolation can be reduced, while social connection, mutual support, and civic responsibility are strengthened. These models emphasize community capacity building alongside clinical care, underscoring that well-being at the end of life is shaped as much by cultural meaning and relationships as by symptom control alone.

Policy frameworks can play a critical role in enabling this shift. Incentivizing partnerships between health systems and local organizations can help integrate medical care with social and spiritual supports, particularly in underserved or rural settings. Public education initiatives that normalize conversations about death and caregiving across the life course may further reduce avoidance and crisis-driven decision-making. Investment in volunteer training, lay navigators, and community connectors can extend the reach of formal palliative services and support family caregivers, fostering resilience within communities while easing pressure on overstretched health systems.

### Conclusion

The UN Decade of Healthy Aging represents an unprecedented opportunity to align aging and dying within a single continuum of well-being. By treating system-supported, goal-aligned end-of-life care as an outcome of integrated life-course care, health systems can move from reactive, disease-oriented care toward anticipatory, equitable models. Integrating palliative principles early across the life course benefits individuals, families, and societies. It affirms that living well and experiencing system-supported, goal-aligned end-of-life care are not opposites but complementary expressions of human flourishing. Future research should develop and validate healthy aging and dying combined metrics, test system-level interventions, and embed these in global healthy aging agendas. Reframing death within health may be one of the most transformative acts of this century.

## Data Availability

Data sharing is not applicable to this article because no datasets were generated or analyzed during the current study.
